# Mindfulness and Perceived Stress: A Study Among Tunisian University Students

**DOI:** 10.1192/j.eurpsy.2025.1481

**Published:** 2025-08-26

**Authors:** A. Guedria, A. Beji, N. Najjar, R. Ayoub, H. Ben Abid

**Affiliations:** 1 Child and Adolescent Psychiatry Departement, Fattouma Bourguiba Hospital; 2 University of Monastir, Monastir; 3 Faculté des Sciences Humaines et Sociales de Tunis, Tunis, Tunisia

## Abstract

**Introduction:**

Mental health among young people, particularly students, has become an increasing concern in contemporary society, shaped by its complexity, fast-paced nature, and hyper-connectivity. High levels of stress have been observed in numerous studies of this population. Perceived stress goes beyond merely observing stressful events, encompassing the individual’s subjective evaluation of these situations. This perception involves awareness of one’s emotions and thoughts, as well as awareness of the surrounding environment.

**Objectives:**

The aim of this study was to explore the relationship between perceived stress and mindfulness levels among Tunisian students.

**Methods:**

This was a cross-sectional study, conducted over a five-month period from August to December 2023. A questionnaire was distributed via email to 800 students, collecting sociodemographic data as well as two scales. The Perceived Stress Scale (PSS-10) was used to evaluate how participants perceive daily life situations as stressful: a higher PSS-10 score indicates greater perceived stress, categorized into three levels: low, moderate, and severe. The Five Factor Mindfulness Questionnaire-15 (FFMQ-15) assessed mindfulness dimensions: observing, describing, acting with awareness, non-judging, and non-reactivity. A higher FFMQ-15 score indicates greater mindfulness.

**Results:**

A total of 102 students agreed to participate in the study. Their ages ranged from 19 to 30, with more than half being under 20 years old. 75.5% of participants were female.

The average PSS-10 score was 23, with significantly higher scores observed among women (p < 10^-3^). Stress levels were mostly moderate (55.88%) or high (32.35%).

The mindfulness assessment revealed that the majority of participants had a moderate level of mindfulness (46 ± 8). Mindfulness scores were higher among students aged between 20 and 30 compared to those under 20 years old (p < 10^-3^).

A negative correlation was observed between perceived stress and mindfulness (p = 0.01; r = -0.508), particularly in three mindfulness dimensions: describing, non-judging, and acting with awareness.

**Conclusions:**

Mindfulness appears to play a significant role in reducing perceived stress levels. Our findings suggest that mindfulness training should be integrated into programs aimed at improving stress management among students.

**Disclosure of Interest:**

None Declared

